# Differences in Bacterial Community Structure on *Hydrilla verticillata* and *Vallisneria americana* in a Freshwater Spring

**DOI:** 10.1264/jsme2.ME13064

**Published:** 2014-02-19

**Authors:** Nadine Gordon-Bradley, Despoina S. Lymperopoulou, Henry Neal Williams

**Affiliations:** 1 School of the Environment Science, Florida A&M University, 1515 S Martin Luther King Jr Blvd Room 305-D, Tallahassee, FL 32307, USA

**Keywords:** *Hydrilla*, *Vallisneria*, bacteria, community structure, clone library

## Abstract

The phylogenetic composition of the epiphytic bacterial community of an invasive aquatic plant (*Hydrilla verticillata*) and a native species (*Vallisneria americana* [eelgrass]) of the Wakulla Spring (Florida) was investigated, along with the water column bacterial composition, using clone libraries of the 16S rRNA genes. The bacterial clones from three clone libraries were classified into 182 operational taxonomic units (OTUs), most of which were affiliated with bacterial divisions commonly found in freshwater ecosystems. Based on the identified classes, the bacterial communities on eelgrass and *Hydrilla* were distinct, such that *Planctomycetes*, *Cyanobacteria*, *Bacilli* and *Actinobacteria* were found on eelgrass and in the water column but not on *Hydrilla*. On the other hand, *Deltaproteobacteria* and *Verrucomicrobiae* were found on *Hydrilla* and in the water column but not on eelgrass. Further distinctions observed were that *Armatimonadia* and *Deinococci* were found only on *Hydrilla* while *Gemmatimonadetes* was found only on eelgrass. Our results indicated differences between the epiphytic bacterial community on the two plants and the water column at the species level, but an even representation of the most abundant phylogenetic taxa (classes) in all three libraries was revealed. Statistical comparison of the retrieved sequences confirmed that the three libraries did not differ significantly at the community level (LIBSHUFF, *p* <0.05).

*Hydrilla* (*Hydrilla verticillata*, [L.F. Royle]), a known global species (25) and native to the warm water regions of Asia (17), is a major invasive aquatic plant and one of the most studied submerged vascular plants. The predominance of *Hydrilla* in the macrophytic communities of infested water bodies is attributed to its mat-forming tendency, the absence of indigenous biological control agents and the relatively low light levels needed to achieve half-maximal photosynthetic rates (12).

*Hydrilla* was first revealed in the USA around 1960 at two sites in Florida, a canal near Miami and in Crystal River (3, 25). Due to its rapid rate of spread (25), by the 1970s *Hydrilla* was distributed throughout the state of Florida (USA), affecting most major freshwater bodies of all drainage basins (25). The invasive plant has become a major problem in water bodies (3, 25) such as Wakulla Spring, which is part of the longest and deepest known submerged freshwater cave systems in the world (9) and is classified as a natural treasure. In Wakulla Spring, dense stands of *Hydrilla* have crowded out *V. americana*, the predominant native submerged plant, causing its decline and in some locations complete displacement (43). This occurs because canopy-forming species such as *Hydrilla* reduce light to deeper waters and sediments where the major portion of the *V. americana* shoot biomass is distributed (1).

The loss of *V. americana*, commonly called eelgrass, tape grass or vallis, can result in profound shifts in fauna, including commercial and recreational species. Freshwater eelgrass is a dioecious, perennial, aquatic bottom-rooted submerged (27) macrophyte (20) that produces long ribbon-like, lightgreen leaves varying in length (2 m or more) based on the depth and movement of the water (20). Eelgrass beds have ecological significance as they provide refuge, feeding grounds, or habitats to many animals (24), including manatees and various species of fish.

In addition, *Hydrilla* destroys native fish and wildlife habitats, out-competes indigenous species, resulting in biodiversity reduction (2), alters nutrient cycling and possibly changes habitat structure (13). Whether *Hydrilla* invasion alters the bacterial community, which plays major roles in ecosystem function has not been widely addressed. According to several reports (10, 42), epiphytic bacterial communities tend to vary based on the plant species. A change in the plant species in an aquatic system could possibly result in a shift in the bacterial community structure of the system. Since bacteria play a vital role in biogeochemical cycles, a change in their community structure could alter certain functions in an entire ecosystem. It has been reported that plant leaf surfaces may serve as hot spots for bacteria since they are able to obtain their supply of carbon from plant exudates (23). Epiphytic bacteria found on aquatic plants are reported to be high in abundance (30, 32) and productivity (4, 23) and are generally larger than free-living bacteria (22). These epiphytic bacteria are likely to contribute significantly to bacterial activity in the ecosystem (41).

Therefore, the replacement of native plant species by the invasive *Hydrilla* has the potential to harbor and/or introduce a new community of bacteria which may impact the bacterial community structure of the water column and result in ecological changes in the spring ecosystem. On the other hand, both *Hydrilla* and eelgrass belong to the same family, *Hydrocharitaceae*, and studies have demonstrated that close plant phylogeny is usually correlated to phylogenetically similar bacterial community structure (33). Thus, to address this potential, the aim of this study was to compare and show distinctions between the bacterial communities of the phyllosphere of the invasive *Hydrilla* and the native eelgrass and the surrounding spring water to assess evidence that the presence of *Hydrilla* contributes to changes in the bacteria community structure of the spring water column.

## Materials and Methods

### Sample collection

The study site for this investigation was Wakulla Spring, located in Wakulla County State Park, Florida, USA. The spring is a first-magnitude spring discharging water at an average of 16.9 m^3^ s^−1^ into the Wakulla River, which flows 14 km to the southeast where it joins St. Mark’s River, which empties into the Gulf of Mexico. The Wakulla Spring bowl is approximately 0.000012 km^2^ while the Wakulla River is approximately 0.29 km^2^. The Wakulla Spring/River is protected in a 24. 28 km^2^ preserve known as the Edward Ball Wakulla Springs State Park (34). Since 1997, the spring has been infested with *Hydrilla verticillata*. During *Hydrilla* bloom, the spring is approximately 70–80% covered with *Hydrilla*.

**Fig. 1:**
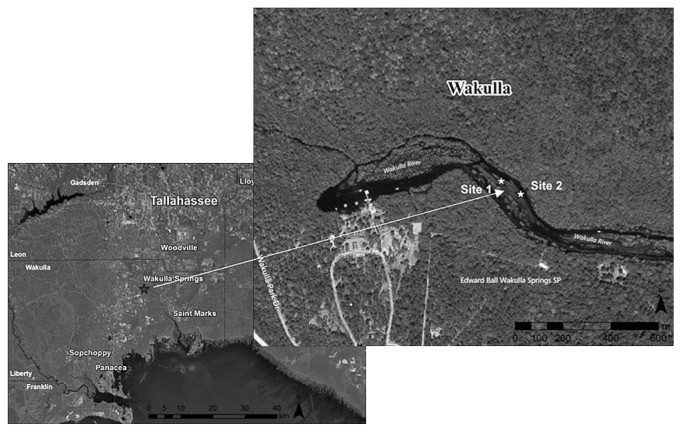
Wakulla Spring with sampling sites.

For this study, two sites were selected during spring 2012 within the Wakulla Spring/River area, Site 1 (N30°14.155′, W084°17.747′) and Site 2 (N30°14.162′, W084°17.777′) (Fig. 1). The two sites selected have similar nutrient availability and water variables (Table 1). They are very close in proximity (approximately 100 m apart) and were two of the regular sampling sites for other objectives of an ongoing study. The water temperature of the spring was measured on site using a YSI instrument (YSI Incorporated, Yellow Springs, OH), while water pH (AR15-Fisher Scientific, Pittsburg, PA) was determined in the laboratory.

Leaves and stems from *Hydrilla*, as well as leaves from eelgrass plants, were collected along with 2 L water samples in duplicate from the two selected sites in the spring. The plant parts were gathered using a mesh that traps the upper sections of eelgrass and *Hydrilla*. Plants were transferred to sterile plastic bags containing spring water from the collection site. Water samples were collected in sterile bottles using a Vertical Point Water Sampler (Aquatic Research Instrument, Hope, ID). In addition, water samples for nutrient analysis were collected for subsequent delivery to the City of Tallahassee Water Quality Laboratory (Tallahassee, FL) for analyses following various EPA methods (Chlorophyll by SMI0200H, Nitrogen Nitrite plus Nitrate by EPA 353, Total phosphorus as P by EPA 365.4 and Organic Carbon Dissolved by SM5310B). Water and plant samples were immediately transported on ice to the laboratory where leaves and stems of *Hydrilla* and leaves of eelgrass were rinsed 3 times with sterilized water to remove loosely attached bacteria and then sectioned into small pieces (approximately 1.2 cm). Twenty grams of each plant sample (wet weight) were stored at −20°C for subsequent DNA extraction. Three liters of composited water samples from both sites were filtered through 0.2 μm Nucleopore track-etched membrane filters (Whatman Laboratory, NJ) to capture bacteria and the filters were stored at −20°C for subsequent DNA extraction.

### DNA extraction, amplification and cloning

DNA was extracted from the *Hydrilla* and eelgrass plant parts following the protocol of Burke *et al.* (8) with some modification. Briefly, 20 g plant material from *Hydrilla* and eelgrass from each site were placed in 500 mL sterilized water (in triplicate) and incubated at room temperature for 2 hours while shaking at 80 rpm to dislodge attached bacteria. Following incubation, plants parts were removed and the remaining water was filtered through 0.2 μm Nucleopore track-etched membrane filters (Whatman Laboratory). Community DNA was extracted from the 0.2 μm filters using the MoBio PowerWater DNA Isolation Kit (MoBio, Carlsbad, CA) following the manufacturer’s instructions. Total DNA was also extracted directly from 200 mg of the leaves and stems of *Hydrilla* and leaves of eelgrass (kept at −20°C) in duplicate using the MoBio PowerPlant Pro DNA Isolation Kit (MoBio) and also from the filter membranes containing bacterial cells from the original water samples using the MoBio PowerWater DNA Isolation Kit (MoBio) according to the manufacturer’s instructions.

Universal eubacterial primers 27F (5′-AGAGTTTGATCCTGG CTCAG-3′ and 1492R (5′-TACGGCTACCTTGTTACGACTT-3′ (35) were used to amplify 16S rRNA gene fragments. The polymerase chain reaction (PCR) mixture was prepared using *Taq* Mastermix (Denville Scientific, Metuchen, NJ) with 2 μL template DNA, PCR grade sterile water and 5 pmol μL^−1^ of each primer in a total reaction fluid of 25 μL. Sterile water was used as negative controls in each batch of PCR reactions. The PCR reactions were performed in a Biorad thermocycler (Hercules, CA) under the following conditions: initial denaturation at 94°C for 5 min, followed by 30 cycles at 94°C for 1 min, annealing at 55°C for 1 min, 72°C for 1 min and a final extension at 72°C for 10 min. Product size and purity were confirmed by electrophoresis in 1% agarose gels stained with ethidium bromide. PCR products were composited to generate 2 clone libraries (*Hydrilla* and eelgrass). A third clone library was generated from the DNA extracted from the water sample. Composited PCR products were purified using MoBio UltraClean PCR Clean-up DNA Purification Kit (MoBio) and cloned into pCR-XL-TOPO vector using the TOPO XL PCR cloning kit (Invitrogen, Carlsbad, CA) in accordance with the manufacturer’s instructions. For each clone library, randomly selected clones containing the insert of the appropriate length (~1,500 bp) were grown in liquid LB medium with kanamycin and their plasmids were purified using the MoBio UltraClean Standard Mini Plasmid Prep Kit for DNA sequencing (MoBio). Purified plasmids were amplified with the cloning kit M13F and M13R primers to confirm the presence of the insert. PCR products were purified with MoBio UltraClean PCR Clean-up DNA Purification Kit (MoBio) and sequenced by the DNA Sequencing Laboratory at Florida State University using M13F and M13R primers. Forward and reverse sequence reads were obtained for each clone and assembled manually using the ClustalW aligning utility tool (http://www.ebi.ac.uk/Tools/clustalw2/index.html), after manually removing vector contamination. The final length of each clone was approximately 1,400 bp. The sequences were screened for chimeras using the DECIPHER program (http://decipher.cee.wisc.edu/FindChimeras.html). Chimerical sequences were excluded from further analysis.

### Phylogenetic analysis

Phylogenetic analysis was performed using the mothur pipeline v. 1.26.0 by Schloss *et al.* (38). Cleaned sequences were aligned using the Silva reference database via the mothur alignment command. Aligned sequences were screened and filtered to remove problematic sequences and sequences classified as chloroplasts. DNA distance matrices were calculated and used to define the number of operational taxonomic units (OTUs) at sequence divergences of 3% (38). All OTUs were compared with GenBank entries using BLAST (http://www.ncbi.nlm.nih.gov/blast) to find the nearest relatives. Phylogenetic trees were constructed from the combined clone libraries with reference sequences from GenBank by the neighbor-joining algorithm based on distances calculated by the Jukes-Cantor model and implemented in the MEGA4 (Molecular Evolutionary Genetics Analysis) software package (40). To confirm the robustness of the tree, bootstrapping was performed with 1,000 replications. Sequences shorter than 1,000 bp were excluded from phylogenetic analysis. OTU-based analysis was performed to determine the number of OTU, Shannon index, chao1 estimates and library coverage. Dendrogram using the thetaYC calculator was generated to determine similarities among the bacterial communities in the clone libraries and LIBSHUFF was used to test comparisons among all the OTUs from all three libraries at 97% similarity. A Venn diagram was constructed to visualize the comparisons and overlaps.

The 16S rRNA gene sequences generated in this study have been deposited in GenBank under accession numbers KC189621– KC189809.

## Results

### Water variables

Water physical and chemical variables (Table 1) were similar at the two sites except for the chlorophyll-a concentration. The reason for this difference is not known because both sites share similar characteristics and are close in proximity. However, based on a report by the Florida Department of Environmental Protection, the chlorophyll-a concentration in the spring occurs over a wide range of 1 μg L^−1^–7.2 μg L^−1^ (16).

### Richness and evenness of the bacterial communities

The richness (Chao1), diversity indices (Shannon), and biodiversity coverage of the clone libraries are shown in Table 2. Similarity (97%) analysis revealed 182 OTUs (54 from the *Hydrilla* library, 48 from the eelgrass library, and 80 from the water library) (Table 2). The Shannon diversity index ranged from 3.7–4.1 with the water column having the highest values. The Chao1, a nonparametric richness estimate of true species diversity in a sample, ranged from 151–335 (Table 2) with eelgrass having the highest value. Coverage analyses indicated that the libraries contained 34% to 58% (Table 2) of the total number of OTUs in the samples.

### Phylogenetic analysis

The construction of clone libraries to determine the composition of bacterial assemblages on leaves and stems of aquatic plants and water samples yielded 143, 145 and 118 clones from *Hydrilla*, water and eelgrass, respectively, from the two combined sampling sites in Wakulla Spring. Following chimera and chloroplast removal, 96, 125 and 64 clones of *Hydrilla*, water and eelgrass, respectively, were subjected to further analysis.

**Fig. 2:**
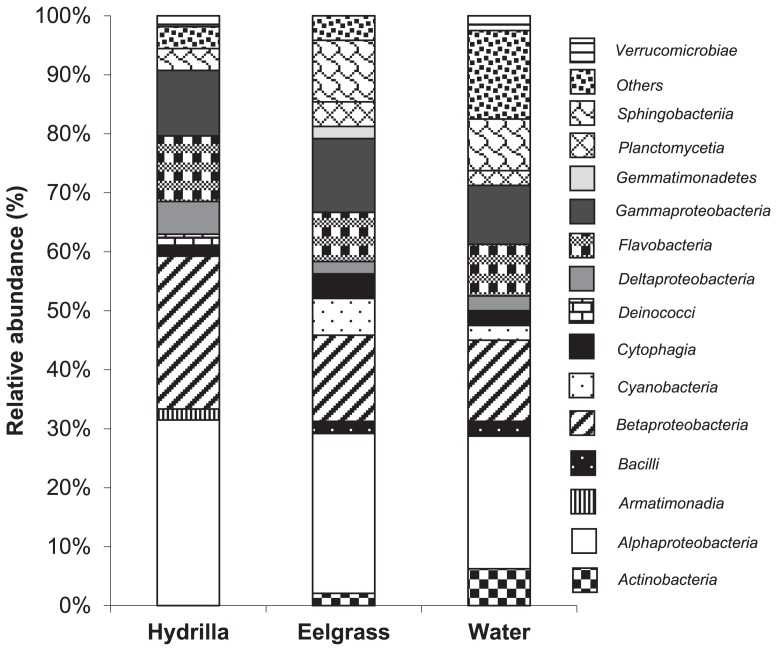
Relative abundance of different bacterial classes found in the clone libraries of *Hydrilla*, eelgrass and water collected from Wakulla Spring.

The composition of the clone libraries is shown in Fig. 2, Fig. S1–S4, and Table S1. Based on the 182 OTUs retrieved across the three libraries (Table 2), 15 known classes were identified (Fig. 2). Phylogenetic analysis showed that most of the commonly identified freshwater bacterial classes were present in the two plant species and the surrounding water column. The *Alphaproteobacteria*, *Betaproteobacteria*, *Gammaproteobacteria*, *Sphingobacteria*, and *Flavobacteria* were the dominant classes in all of the three clone libraries; *Deltaproteobacteria* and *Cytophagia* were also present in all three samples but in much lower abundance. The term “abundance” is used in an abstract sense and refers to relative abundance of ribotypes (16S rRNA gene OTUs) and not to cell abundance.

*Planctomycetes*, *Cyanobacteria*, *Bacilli*, and *Actinobacteria* were detected in the eelgrass and water clone libraries but not in the *Hydrilla* library. *Verrucomicrobiae* were retrieved from both the *Hydrilla* and water column clone libraries but not from the eelgrass. *Deltaproteobacteria* were also only detected in *Hydrilla* and water. *Armatimonadia* and *Deinococci* were present only in the *Hydrilla* clone library, while *Gemmatimonadetes* was detected only in the eelgrass clone library.

At the genus level, the identified genera differed among the three libraries. *Flavobacterium* and *Rhodobacter* were the only common genera found among the libraries (Table 3). *Rhodobacter* and *Flavobacterium* were also the most abundant genera in both eelgrass and *Hydrilla* clone libraries, while *Rhodobacter* and *Ilumatobacter* were the most abundant in the water.

### Library comparisons

**Fig. 3:**
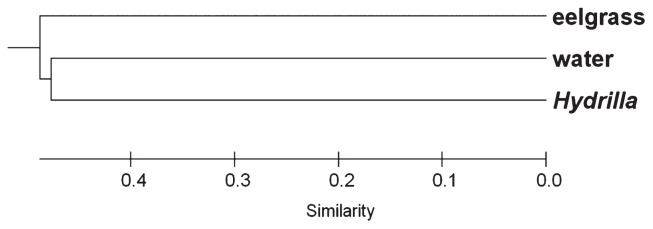
Cluster analysis of the bacterial community in the water column and the epiphytic bacteria on aquatic plants from Wakulla Spring.

**Fig. 4:**
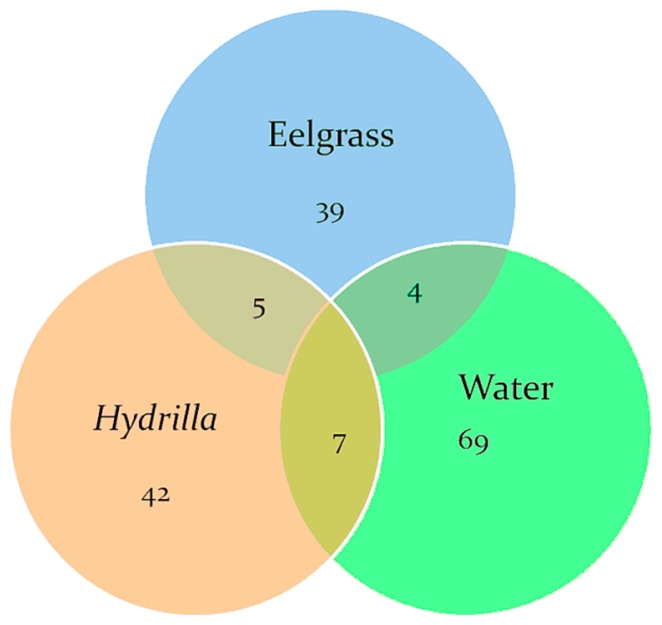
Venn diagram of the bacterial OTUs detected on samples of the epiphytic communities from eelgrass and *Hydrilla* and the water in Wakulla Spring. Numbers in the diagram represent the OTUs retrieved from each library with those in the overlapped areas shared between the overlapping libraries.

Similarity in the community structure from *Hydrilla*, eelgrass, and the water column were measured using thetaYC calculator in mothur (Fig. 3). The results from the library comparison showed low similarity among the three clone libraries based on the relative abundance of OTUs in each library; however, the *Hydrilla* bacteria appeared to be more closely related to the water column bacteria than to eelgrass bacteria. Nevertheless, LIBSHUFF analysis showed that the three clone libraries were not significantly different (Table 4). No OTUs (Fig. 4) common to all three samples (two plants and water) were detected.

## Discussion

### Richness and evenness of the bacterial communities

The Shannon diversity index showed the water column to have the greatest diversity and abundance of bacteria. This should not be surprising because water is more likely to accommodate a higher diversity of bacteria due to the many various sources through which bacteria can be introduced into the spring. In contrast, the bacterial community on the phyllosphere of the two plants is expected to be more established and stable (11). Bacteria from the native eelgrass had the highest Chao1 richness estimate, exhibiting higher species richness than the *Hydrilla* and the water column communities. Chao1 takes into account the number of OTUs that appear either once or twice in a library. In our study, the eelgrass library was under-sampled (only 34% coverage) compared to the water and the *Hydrilla* libraries (52% and 58%, respectively), mainly due to the removal of many chloroplast-like sequences. Although the number of clones screened and sequenced did not represent a full inventory of those present on the plants and water samples based on the findings from this and previous studies (18, 29, 44, 45), the coverage was adequate to provide a full inventory of the dominant species.

### Phylogenetic analysis

Phylogenetic analysis showed *Alphaproteobacteria*, *Betaproteobacteria*, *Gammaproteobacteria*, *Sphingobacteria*, and *Flavobacteria* as the dominant classes in each of the three clone libraries, while *Deltaproteobacteria* and *Cytophagia* were also present in all three samples but in much lower abundance. These findings are consistent with the results reported by Crump and Koch (10) who found the phylum *Proteobacteria* to be dominant on leaf surfaces of angiosperms from Chesapeake Bay. Buesing *et al.* (7) found *Alphaproteobacteria* to be the second most abundant group of bacteria after the CFB group on submerged macrophytes in brackish water.

In a study similar to ours in Lake Taihu, He *et al.* (18) found a high abundance of *Cyanobacteria* on both eelgrass and *Hydrilla* whereas in our study, *Cyanobacteria* was found in low abundance on eelgrass and was completely absent on *Hydrilla*. Also, rare freshwater phyla such as *Gemmatimonadetes*, *Armatimonadia*, *Cytophagia*, *Deinococci*, and *Verrucomicrobia*, were not detected in Lake Taihu. The differences between the epiphytic bacterial communities found in Taihu and Wakulla Spring could be due to geographic location and individuality of the plant species studied.

Other known aquatic classes such as *Planctomycetes*, *Cyanobacteria*, *Bacilli*, and *Actinobacteria* were found only in the eelgrass and water clone libraries, but not in the *Hydrilla* library. Among them, *Planctomycetes* are found in a wide variety of habitats (6) of diverse tropic statuses (37) and are known to be epiphytic (6).

*Deltaproteobacteria* was found only in the water and *Hydrilla* libraries, but not in eelgrass, as was the *Verrucomicrobiae. Verrucomicrobiae* are reported to be active polysaccharide degraders in freshwater environments (28). Boucheret *et al.* (5) reported that the *Verrucomicrobia* serve functions similar to those of *Bacteroidetes*, which are known to degrade biopolymers such as cellulose and chitin (21). Lindström *et al.* (26) found that they favored increased phosphorus availability. Such correlations between the retrieved taxa and observed ecological functions here and throughout the discussion are used to elucidate the potential role of these taxa in the ecosystems but by no means do they imply that such functions are carried out in the Wakulla Spring system, as we did not conduct any functional analysis.

Other rare taxa found sporadically either in the *Hydrilla* or in the eelgrass clone library were *Armatimonadia* and *Deinococci*, and *Gemmatimonadetes*, respectively. Although their functions were not studied in this investigation, rare taxa can be important to ecosystem functions (15).

At the genus level, *Flavobacterium* and *Rhodobacter* were the only two shared genera among all libraries. *Rhodobacter* has been associated with active denitrification in the presence of sulfide-free flow water in the Kama River (36) and is commonly found in freshwater. *Flavobacteria* are also wellknown inhabitants of freshwater systems and are reported to carry out functions such as degradation of complex biopolymers in lake ecosystems (31).

Differences observed in the bacterial taxa between the *Hydrilla* and eelgrass could be due to variations in the types of plant exudates produced (39). Varieties of plants are reported to contain different leaching metabolites and proportions of chemicals (14). It has been reported that *Hydrilla* contains compounds such as loliolide, thymidine, octadecanedioic acid (46) and caffeic acid ester (19). Whether the bacteria can utilize these carbon compounds as energy sources is not fully understood; however, this could be an important area for future investigation and contribute to knowledge on the factors that govern the colonization of *Hydrilla* and eelgrass surfaces.

Differences in the leaf structure of eelgrass and *Hydrilla* may also affect the bacterial community on them. *Hydrilla* has small, strap-like leaves that are pointed and grow in whorls of four to eight around the stem, which may provide a more protective habitat for bacterial colonizers. Eelgrass has a broad, straight, flat leaf surface ranging up to 25 mm in width, which could be a challenging habitat for some bacteria to colonize, especially those that have not evolved to survive on surfaces exposed to constant flowing water such as in Wakulla Spring.

Although plant-host specificity is crucial for the structure of the epiphytic bacterial community, the role of the surrounding water is still understudied. Both *Hydrilla* and eelgrass co-exist in the same habitat, and the samples were taken from plants in close proximity (100 m). The exposure of both plants to the same bacterial milieu (water) has the potential to render all three communities phylogenetically similar (33). In addition, the close phylogenetic relationship of *Hydrilla* and eelgrass raises the expectation that they may harbor similar bacterial communities at the phylogenetic level (33). However, other special characteristics of each plant such as leaf structure and exudates could have a greater role in dictating the types of microbes that thrive on them (33) and could account for differences in the species retrieved from the two plants.

In one of the first studies comparing the epiphytic bacterial communities on *Hydrilla verticillata*, eelgrass, and the water column, differences were found at the species level between the epiphytic bacterial community on the two plants and the water column. However, the distribution of bacterial classes was similar in all three communities. Further investigation with a more extensive sampling strategy of the specific phyllosphere bacterial communities on *Hydrilla* and eelgrass is encouraged to confirm the results from this and other studies (18) and to provide greater insights into the ecological impacts of *Hydrilla*’s introduction into aquatic systems. Also, further work on the chemical composition of *Hydrilla*, eelgrass, and other aquatic plant species may provide additional information as to the factors that select the bacterial communities on the two plant species. The results of this study serve as a segue to future studies in this area.

## SUPPLEMENTARY MATERIAL



## Figures and Tables

**Table 1:** Measurements of water physical and chemical variables at the two sampling sites in Wakulla Spring

Water Environmental Variables	Site 1	Site 2
Temperature (°C)	21.3	21.3
pH	8.33	8.30
NH_3_-N(mg L^−1^)	0.50	0.56
Total phosphate (mg L^−1^)	0.02	0.02
Chlorophyll-a (μg L^−1^)	5.90	7.40
Dissolved organic carbon (mg L^−1^)	0.35	0.35

**Table 2:** Comparison of bacterial operational taxonomic units (OTUs) among clone libraries as determined by mothur pipeline

	Shannon	Chao1	Coverage	# of clones	# of OTUs
*Hydrilla*	3.6	151	58%	96	54
Eelgrass	3.7	335	34%	64	48
Water	4.1	206	52%	125	80

**Table 3:** Bacterial classes and genera identified among the plant species and the water column of Wakulla Spring in Wakulla County, Florida. Key: “+” = present, “−” = absent

Bacterial class/Genera	Eelgrass	Hydrilla	Water
***Class; Gemmatimonadetes***	+	−	−
*Gemmatimonas*	+	−	−
***Class; Armatimonadia***	−	+	−
*Armatimonas/Armatimonadetes_gp1*	−	+	−
***Class; Cytophagia***	+	+	+
*Flexibacter sp.*	−	+	−
***Class; Deinococci***	−	+	−
*Truepera*	−	+	−
***Class; Betaproteobacteria***	+	+	+
*Undibacterium*	+	−	+
*Hydrogenophaga*	+	−	−
*Acidovorax*	−	+	+
*Inhella*	−	+	−
*Limnobacter*	−	+	−
*Limnohabitans*	−	−	+
*Polynucleobacter*	−	−	+
***Class; Alphaproteobacteria***	+	+	+
*Methylophilus*	−	+	−
*Sphingopyxis*	−	−	+
*Rhizobium*	+	−	+
*Porphyrobacter*	+	−	−
*Rhodobacter*	+	+	+
*Sandarakinorhabdus*	+	−	−
*Novosphingobium*	−	+	−
*Sphingomonas*	−	+	+
*Devosia*	−	−	+
***Class; Gammaproteobacteria***	+	+	+
*Coxiella*	+	−	−
*Pseudomonas*	−	+	+
*Cellvibrio*	−	+	+
*Steroidobacter*	−	+	−
*Legionella*	−	−	+
*Vibrio*	−	−	+
***Class; Sphingobacteria***	+	+	+
*Leadbetterella*	−	−	+
*Arcicella*	+	−	−
*Haliscomenobacter*	+	−	+
*Ferruginibacter*	+	−	−
*Chitinophaga*	−	−	+
***Class; Planctomycetia***	+	−	+
*Planctomyces*	−	−	+
***Class; Actinobacteria***	+	−	+
*Ilumatobacter*	+	−	+
***Class; Cyanobacteria***	+	−	+
*Gp1*	+	−	−
***Class; Flavobacteria***	+	+	+
*Flavobacterium*	+	+	+
***Class; Verrucomicrobia***	−	+	+
*Prosthecobacter*	−	−	+
*Luteolibacter*	−	−	+
***Class; Bacilli***	+	−	+
*Pasteuria*	−	−	+
***Class; Deltaproteobacteria***	−	+	+
*Bdellovibrio*	−	+	−

**Table 4:** Values in the table are the probabilities that the compositions of the libraries are different, as calculated using the LIBSHUFF command implemented in MOTHUR and the critical threshold set at 0.05/6=0.0083333 (X compared to Y, Y compared to X, where X is the library indicated in the column stub and Y is the library in the row head).

	eelgrass	water
*Hydrilla*	0.015, 0.004	0.010, 0.020
eelgrass		0.004, 0.020
