# Supplementary material for: Differences in Bacterial Community Structure on Hydrilla verticillata and Vallisneria americana in a Freshwater Spring
Source: Microbes Environ. 2014 Feb 19;29(1):67–73. doi: 10.1264/jsme2.ME13064 (PMC4041241; doi:10.1264/jsme2.ME13064)
Supplement: Supplementary file 1 [file 29_67_s1.pdf]

SUPPLEMENTARY MATERIAL

Gordon-Bradley *et al.*, submited to "Microbes and Environments"

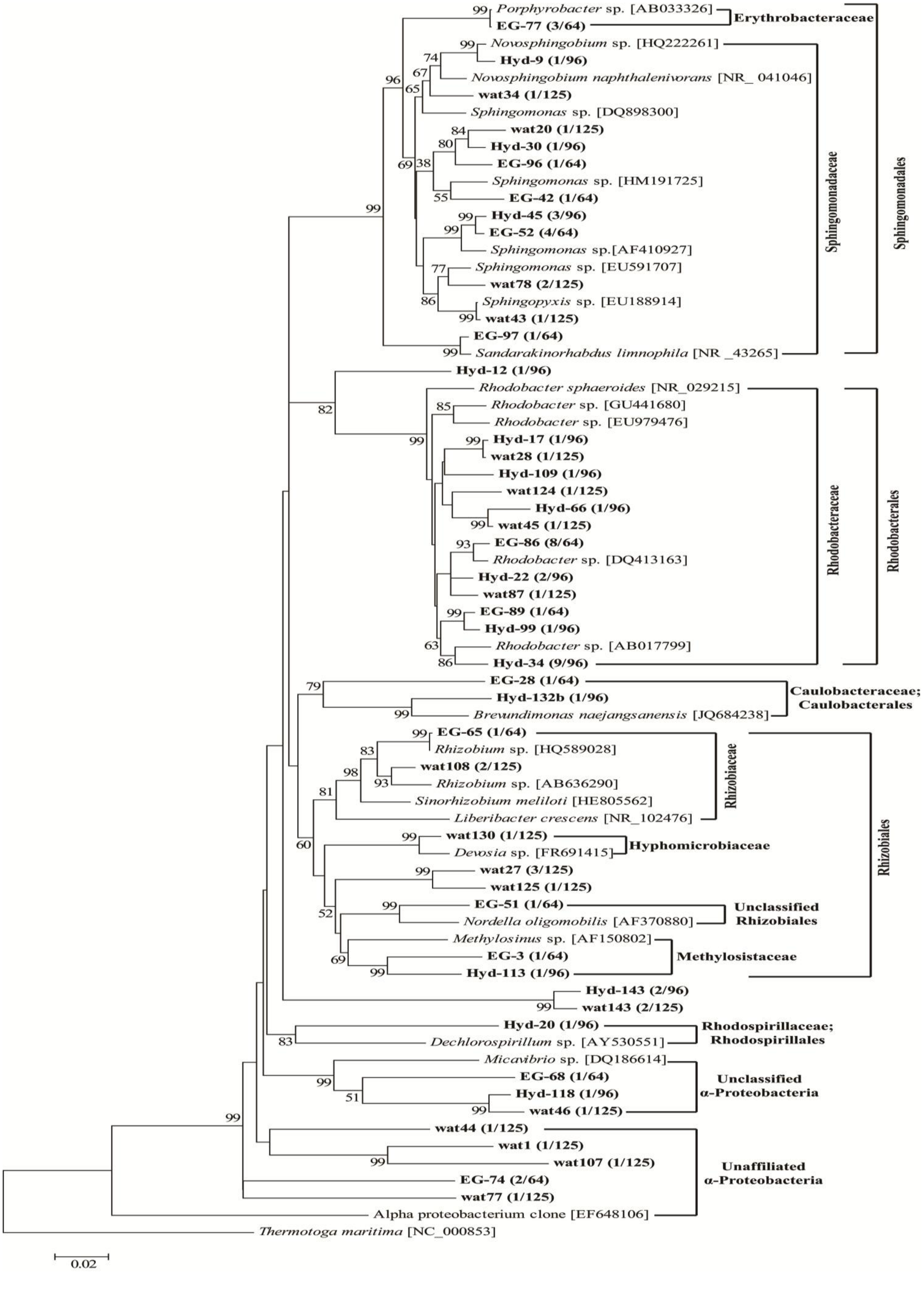

Porphyrobacter sp. [AB033326]
EG-77 (3/64)
Erythrobacteraceae
Novosphingobium sp. [HQ222261]
Hyd-9 (1/96)
Novosphingobium naphthalenivorans [NR_ 041046]
wat34 (1/125)
Sphingomonas sp. [DQ898300]
wat20 (1/125)
Hyd-30 (1/96)
EG-96 (1/64)
Sphingomonas sp. [HM191725]
EG-42 (1/64)
Hyd-45 (3/96)
EG-52 (4/64)
Sphingomonas sp.[AF410927]
Sphingomonas sp. [EU591707]
wat78 (2/125)
Sphingopyxis sp. [EU188914]
wat43 (1/125)
EG-97 (1/64)
Sandarakinorhabdus limnophila [NR _43265]
Sphingomonadaceae
Sphingomonadales
Hyd-12 (1/96)
Rhodobacter sphaeroides [NR_029215]
Rhodobacter sp. [GU441680]
Rhodobacter sp. [EU979476]
Hyd-17 (1/96)
wat28 (1/125)
Hyd-109 (1/96)
wat124 (1/125)
Hyd-66 (1/96)
wat45 (1/125)
EG-86 (8/64)
Rhodobacter sp. [DQ413163]
Hyd-22 (2/96)
wat87 (1/125)
EG-89 (1/64)
Hyd-99 (1/96)
Rhodobacter sp. [AB017799]
Hyd-34 (9/96)
Rhodobacteraceae
Rhodobacterales
EG-28 (1/64)
Hyd-132b (1/96)
Brevundimonas naejangsanensis [JQ684238]
Caulobacteraceae; Caulobacterales
EG-65 (1/64)
Rhizobium sp. [HQ589028]
wat108 (2/125)
Rhizobium sp. [AB636290]
Sinorhizobium meliloti [HE805562]
Liberibacter crescens [NR_102476]
Rhizobiaceae
wat130 (1/125)
Dewosia sp. [FR691415]
Hyphomicrobiaceae
wat27 (3/125)
wat125 (1/125)
EG-51 (1/64)
Nordella oligomobilis [AF370880]
Unclassified Rhizobiales
Methylosinus sp. [AF150802]
EG-3 (1/64)
Hyd-113 (1/96)
Methylosistaceae
Rhizobiales
Hyd-143 (2/96)
wat143 (2/125)
Hyd-20 (1/96)
Dechlorospirillum sp. [AY530551]
Rhodospirillaceae; Rhodospirillales
Micavibrio sp. [DQ186614]
EG-68 (1/64)
Hyd-118 (1/96)
wat46 (1/125)
Unclassified α-Proteobacteria
wat44 (1/125)
wat1 (1/125)
wat107 (1/125)
EG-74 (2/64)
wat77 (1/125)
Alpha proteobacterium clone [EF648106]
Unaffiliated α-Proteobacteria
Thermotoga maritima [NC_000853]
0.02

Supplemental Figure S1 Phylogenetic tree of the PCR-amplified 16S rRNA gene (in bold) (c. 1390 bp) of the α-Proteobacteria in the water column and the two plant (*Hydrilla* and eelgrass) libraries of Wakulla Spring, based on the neighbour-joining method as determined by distance Jukes-Cantor analysis. One thousand bootstrap analyses (distance) were conducted and percentages ≥50% is indicated at nodes. Clones (bold letters) that have ≥97% similar nucleic acid sequences within each sampling site are represented by a single sequence (OTU), with the number of clones out of the total in parentheses. The numbers in brackets are GenBank accession numbers. Scale bar represents 2% estimated distance

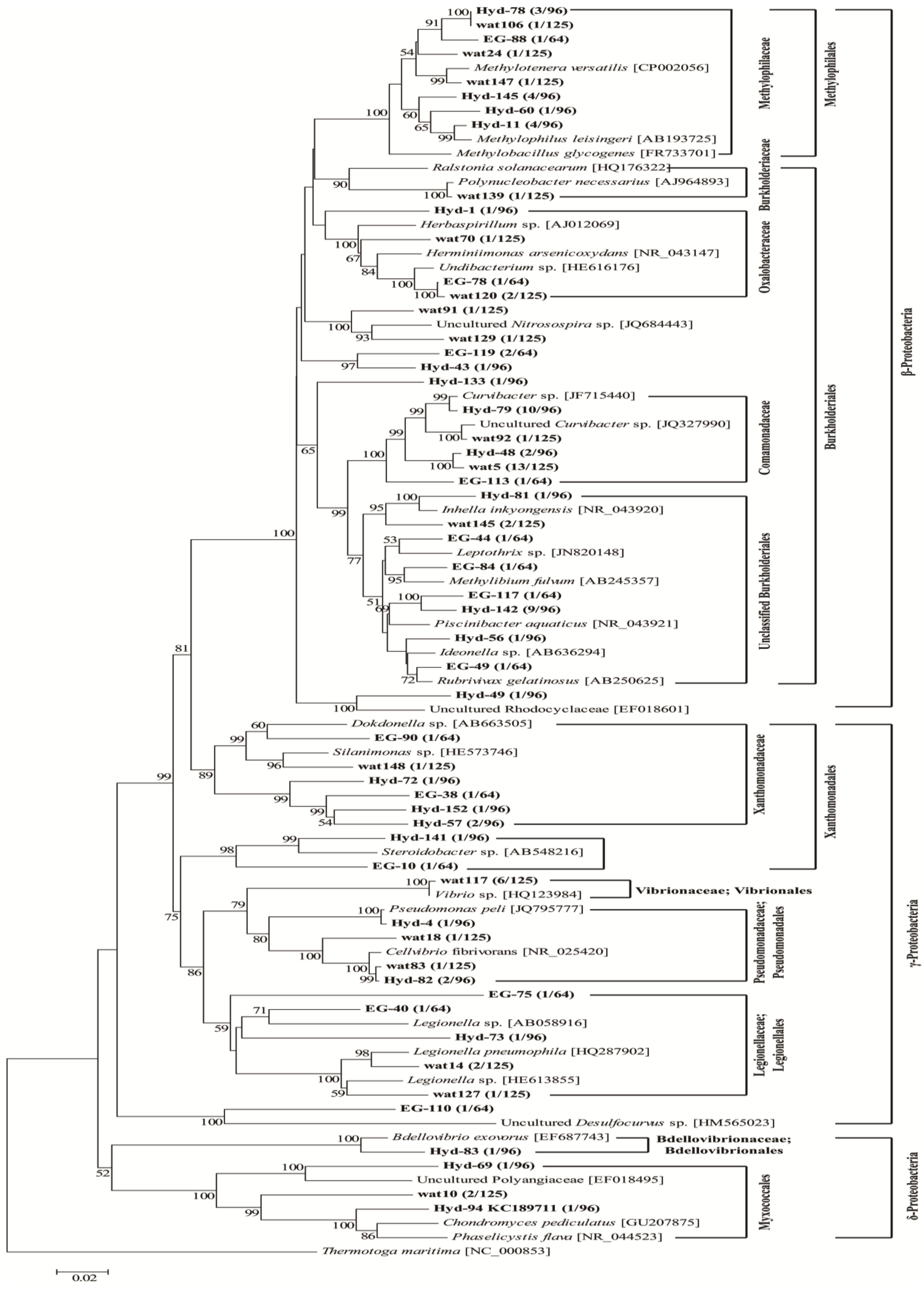

Hyd-78 (3/96)
wat106 (1/125)
EG-88 (1/64)
wat24 (1/125)
Methylotenera versatilis [CP002056]
wat147 (1/125)
Hyd-145 (4/96)
Hyd-60 (1/96)
Hyd-11 (4/96)
Methylophilus leisingeri [AB193725]
Methylobacillus glycogenes [FR733701]
Ralstonia solanacearum [HQ176322]
Polynucleobacter necessarius [AJ964893]
wat139 (1/125)
Hyd-1 (1/96)
Herbaspirillum sp. [AJ012069]
wat70 (1/125)
Herminiimonas arsenicoxydans [NR_043147]
Undibacterium sp. [HE616176]
EG-78 (1/64)
wat120 (2/125)
wat91 (1/125)
Uncultured Nitrosospira sp. [JQ684443]
wat129 (1/125)
EG-119 (2/64)
Hyd-43 (1/96)
Hyd-133 (1/96)
Curvibacter sp. [JF715440]
Hyd-79 (10/96)
Uncultured Curvibacter sp. [JQ327990]
wat92 (1/125)
Hyd-48 (2/96)
wat5 (13/125)
EG-113 (1/64)
Hyd-81 (1/96)
Inhella inkyongensis [NR_043920]
wat145 (2/125)
EG-44 (1/64)
Leptothrix sp. [JN820148]
EG-84 (1/64)
Methylibium fulvum [AB245357]
EG-117 (1/64)
Hyd-142 (9/96)
Piscinibacter aquaticus [NR_043921]
Hyd-56 (1/96)
Ideonella sp. [AB636294]
EG-49 (1/64)
Rubrivivax gelatinosus [AB250625]
Hyd-49 (1/96)
Uncultured Rhodocyclaceae [EF018601]
Dokdonella sp. [AB663505]
EG-90 (1/64)
Silanimonas sp. [HE573746]
wat148 (1/125)
Hyd-72 (1/96)
EG-38 (1/64)
Hyd-152 (1/96)
Hyd-57 (2/96)
Hyd-141 (1/96)
Steroidobacter sp. [AB548216]
EG-10 (1/64)
wat117 (6/125)
Vibrio sp. [HQ123984]
Vibrionaceae; Vibrionales
Pseudomonas peli [JQ795777]
Hyd-4 (1/96)
wat18 (1/125)
Cellvibrio fibrivorans [NR_025420]
wat83 (1/125)
Hyd-82 (2/96)
EG-75 (1/64)
EG-40 (1/64)
Legionella sp. [AB058916]
Hyd-73 (1/96)
Legionella pneumophila [HQ287902]
wat14 (2/125)
Legionella sp. [HE613855]
wat127 (1/125)
EG-110 (1/64)
Uncultured Desulfocurvus sp. [HM565023]
Bdellovibrio exovorus [EF687743]
Hyd-83 (1/96)
Bdellovibrionaceae; Bdellovibrionales
Hyd-69 (1/96)
Uncultured Polyangiaceae [EF018495]
wat10 (2/125)
Hyd-94 KC189711 (1/96)
Chondromyces pediculatus [GU207875]
Phaselicystis flava [NR_044523]
Thermotoga maritima [NC_000853]
0.02
Methylophilaceae
Burkholderiaceae
Oxalobacteraceae
Comamonadaceae
Unclassified Burkholderiales
Xanthomonadaceae
Pseudomonadaceae; Pseudomonadales
Legionellaceae; Legionellales
Myxococcales
Methylophilales
Burkholderiales
Xanthomonadales
β-Proteobacteria
γ-Proteobacteria
δ-Proteobacteria

Supplemental Figure S2 Phylogenetic tree of the PCR-amplified 16S rRNA gene OTUs (in bold) (c. 1390 bp) of the β-, γ- and δ-Proteobacteria in the water column and the two plant (*Hydrilla* and eelgrass) libraries of Wakulla Spring, based on the neighbour-joining method as determined by distance Jukes-Cantor analysis. Numbers of identical (≥97% sequence similarity) OTUs of the total number OTUs found in each station are shown in parentheses. One thousand bootstrap analyses (distance) were conducted and percentages ≥50% is indicated at nodes. Numbers in brackets are GenBank accession numbers. Scale bar represents 2% estimated distance.

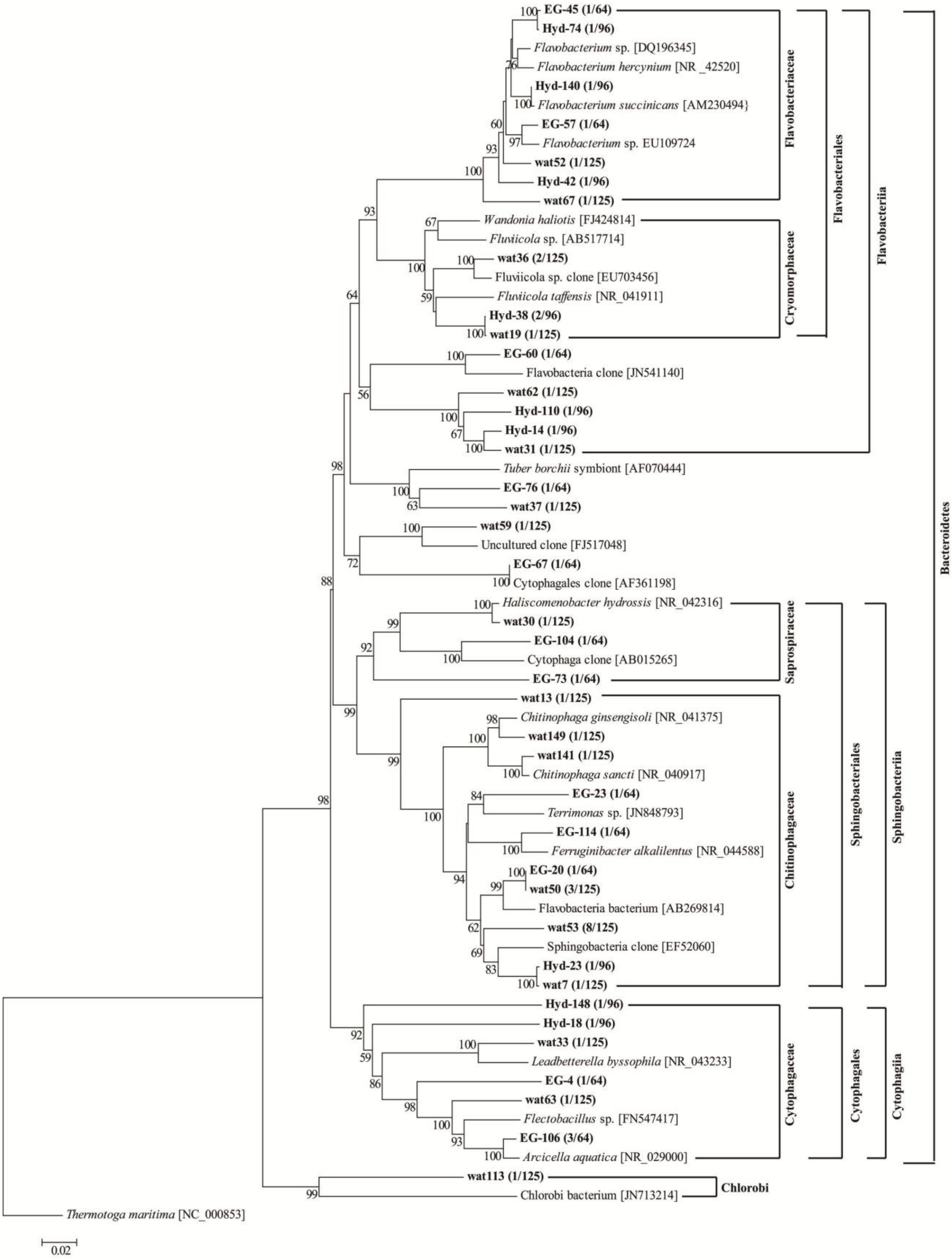
EG-45 (1/64)
Hyd-74 (1/96)
Flavobacterium sp. [DQ196345]
Flavobacterium hercynium [NR_42520]
Hyd-140 (1/96)
Flavobacterium succinicans [AM230494}
EG-57 (1/64)
Flavobacterium sp. EU109724
wat52 (1/125)
Hyd-42 (1/96)
wat67 (1/125)
Wandonia haliotis [FJ424814]
Fluviicola sp. [AB517714]
wat36 (2/125)
Fluviicola sp. clone [EU703456]
Fluviicola taffensis [NR_041911]
Hyd-38 (2/96)
wat19 (1/125)
EG-60 (1/64)
Flavobacteria clone [JN541140]
wat62 (1/125)
Hyd-110 (1/96)
Hyd-14 (1/96)
wat31 (1/125)
Tuber borchii symbiont [AF070444]
EG-76 (1/64)
wat37 (1/125)
wat59 (1/125)
Uncultured clone [FJ517048]
EG-67 (1/64)
Cytophagales clone [AF361198]
Haliscomenobacter hydrossis [NR_042316]
wat30 (1/125)
EG-104 (1/64)
Cytophaga clone [AB015265]
EG-73 (1/64)
wat13 (1/125)
Chitinophaga ginsengisoli [NR_041375]
wat149 (1/125)
wat141 (1/125)
Chitinophaga sancti [NR_040917]
EG-23 (1/64)
Terrimonas sp. [JN848793]
EG-114 (1/64)
Ferruginibacter alkalilentus [NR_044588]
EG-20 (1/64)
wat50 (3/125)
Flavobacteria bacterium [AB269814]
wat53 (8/125)
Sphingobacteria clone [EF52060]
Hyd-23 (1/96)
wat7 (1/125)
Hyd-148 (1/96)
Hyd-18 (1/96)
wat33 (1/125)
Leadbetterella byssophila [NR_043233]
EG-4 (1/64)
wat63 (1/125)
Flectobacillus sp. [FN547417]
EG-106 (3/64)
Arcicella aquatica [NR_029000]
wat113 (1/125)
Chlorobi bacterium [JN713214]
Thermotoga maritima [NC_000853]
Flavobacteriaceae
Cryomorphaceae
Flavobacteriales
Flavobacteria
Saprospiraceae
Chitinophagaceae
Sphingobacteriales
Sphingobacteriia
Cytophagaceae
Cytophagales
Cytophagiia
Bacteroidetes
Chlorobi
0.02

Supplemental Figure S3 Phylogenetic tree of the PCR-amplified 16S rRNA gene OTUs (in bold) (c. 1390 bp) of the Bacteroidetes/Chlorobi group in the water column and the two plants (*Hydrilla* and eelgrass) libraries of Wakulla Spring, based on the neighbour-joining method as determined by distance Jukes-Cantor analysis. Numbers of identical (≥97% sequence similarity) OTUs of the total number OTUs found in each station are shown in parentheses. One thousand bootstrap analyses (distance) were conducted and percentages ≥50% is indicated at nodes. Numbers in brackets are GenBank accession numbers. Scale bar represents 2% estimated distance.

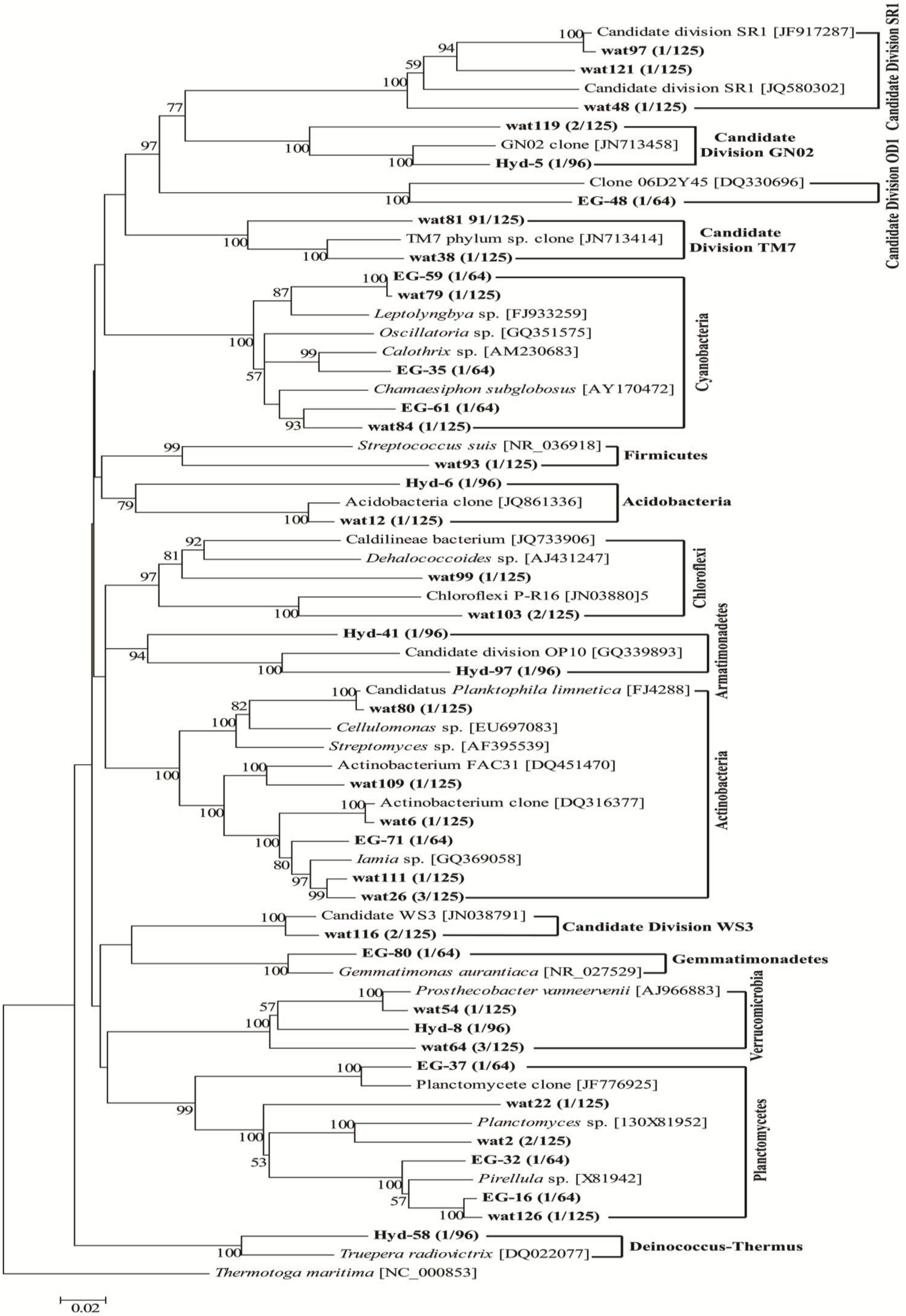
Candidate division SR1 [JF917287]
wat97 (1/125)
wat121 (1/125)
Candidate division SR1 [JQ580302]
wat48 (1/125)
Candidate Division SR1
wat119 (2/125)
GN02 clone [JN713458]
Hyd-5 (1/96)
Candidate Division GN02
Clone 06D2Y45 [DQ330696]
EG-48 (1/64)
Candidate Division OD1
wat81 91/125)
TM7 phylum sp. clone [JN713414]
wat38 (1/125)
Candidate Division TM7
EG-59 (1/64)
wat79 (1/125)
Leptolyngbya sp. [FJ933259]
Oscillatoria sp. [GQ351575]
Calothrix sp. [AM230683]
EG-35 (1/64)
Chamaesiphon subglobosus [AY170472]
EG-61 (1/64)
wat84 (1/125)
Cyanobacteria
Streptococcus suis [NR_036918]
wat93 (1/125)
Firmicutes
Hyd-6 (1/96)
Acidobacteria clone [JQ861336]
wat12 (1/125)
Acidobacteria
Caldilineae bacterium [JQ733906]
Dehalococcoides sp. [AJ431247]
wat99 (1/125)
Chloroflexi P-R16 [JN03880]5
wat103 (2/125)
Chloroflexi
Hyd-41 (1/96)
Candidate division OP10 [GQ339893]
Hyd-97 (1/96)
Armatimonadetes
Candidatus Planktophila limnetica [FJ4288]
wat80 (1/125)
Cellulomonas sp. [EU697083]
Streptomyces sp. [AF395539]
Actinobacterium FAC31 [DQ451470]
wat109 (1/125)
Actinobacterium clone [DQ316377]
wat6 (1/125)
EG-71 (1/64)
Iamia sp. [GQ369058]
wat111 (1/125)
wat26 (3/125)
Actinobacteria
Candidate WS3 [JN038791]
wat116 (2/125)
Candidate Division WS3
EG-80 (1/64)
Gemmatimonas aurantiaca [NR_027529]
Gemmatimonadetes
Prosthecobacter vanneervenii [AJ966883]
wat54 (1/125)
Hyd-8 (1/96)
wat64 (3/125)
Verrucomicrobia
EG-37 (1/64)
Planctomycete clone [JF776925]
wat22 (1/125)
Planctomyces sp. [130X81952]
wat2 (2/125)
EG-32 (1/64)
Pirellula sp. [X81942]
EG-16 (1/64)
wat126 (1/125)
Planctomycetes
Hyd-58 (1/96)
Truepera radiovictrix [DQ022077]
Deinococcus-Thermus
Thermotoga maritima [NC_000853]
0.02

Supplemental Figure S4 Phylogenetic tree of the PCR-amplified 16S rRNA gene OTUs (in bold) (c. 1390 bp) of the rest of the retrieved OTUs in the water column and the two plants (*Hydrilla* and eelgrass) libraries of Wakulla Spring, based on the neighbour-joining method as determined by distance Jukes-Cantor analysis. Numbers of identical (≥97% sequence similarity) OTUs of the total number phylotypes found in each station are shown in parentheses. One thousand bootstrap analyses (distance) were conducted and percentages ≥50% is indicated at nodes. Numbers in brackets are GenBank accession numbers. Scale bar represents 2% estimated distance.

Supplemental Table S1 Occurrence of bacterial OTUs in the water column and the two plants (*Hydrilla* and eelgrass) libraries of Wakulla Spring

Phylotype	No. of similar(≥97%) clones	Putative affiliation	Closest sequence (similarity) [GenBank accession No.]	Description	Closest organism (similarity) [GenBank accession No.]
EG-3	1	Alphaproteobacteria	Clone D10_29(95.37)[EU266798]	quifer sediments	Hyphomicrobium sulfonivorans 25S (93.86)[AY305006]
EG-4	1	Sphingobacteriia	Clone reservoir-75 16S (87.77)[JF697456]	Stream of Dianchi Lake	Flectobacillus sp. WG3(87.09)[FN547417]
EG-10	1	Gammaproteobacteria	Clone V201-189(98.76)[HQ114204]	Biofilms in a vermifilter	Uncultured Pseudomonadales Plot18-A06(94.26)[FJ889291]
EG-16	1	Bacilli	Clone cpw3b516 (96.88)[HM921122]	Groundwater	Pirellula sp. Schlesner 302 (92.05)[X81942]
EG-20	1	Flavobacteriia	Clone M17-10-B17(97.51)[JQ088393]	Crude oil reservoir	Flavobacteria bacterium KF030 (96.05)[AB269814]
EG-22	1	Planctomycetia	Clone HDB_SIPA613 (99.32)[HM186830]	Subsurface Soil	Gemmata sp. Io1-6 (92.24)[GQ889466]
EG-23	1	Sphingobacteriia	Clone B193(2011)(91.17)[JF830232]	Grass carp intestine	Terrimonas sp. YJ03 (91.07)[JN848793]
EG-28	1	Alphaproteobacteria	Clone Dianchi-17 (98.07)[HQ324864]	Lake water	Pleomorphomonas oryzae F-4 (89.08)[AB159681]
EG-32	1	Planctomycetia	Clone T-29(95.99)[HQ661341]	Tingxi freshwater reservoir	Pirellula sp. Schlesner 302 (92.79)[X81942]
EG-35	1	Cyanobacteria	Rivularia sp. MU24 UAM-305 (92.41)[EU009149]	Rock surface	Calothrix sp. BECID33(92.49)[AM230683]
EG-37	1	Planctomycetia	Clone Alchichica_AQ2_1_1B_09 (97.02)[JN825597]	Microbialites in aquarium	Uncultured planctomycete QZ-J25 (93.52)[JF776925]
EG-38	1	Gammaproteobacteria	Clone 2\SC\46 (94.55)[EU340183]	Aquatic macrophytes	Dokdonella sp. TSY06 (90.12)[AB663505]
EG-40	1	Gammaproteobacteria	Clone JH-WH38(98.80)[EF492925]	Soil	Legionella sp. OA32(91.44)[AB058916]
EG-42	1	Alphaproteobacteria	Clone 3BR-10BB (96.62)[EU937905]	Riparian iron oxidizing biofilm	Sphingomonas sp. SH (95.37)[HM191725]
EG-44	1	Betaproteobacteria	Leptothrix mobilis Feox-1 (96.78)[NR_026333]		Leptothrix sp. G6(2012) (96.50)[JN820148]
EG-45	1	Flavobacteriia	Clone 3C003394 (97.56)[EU802008]	Chesapeake Bay, MD	Flavobacterium sp. MSY2 (97.16)[DQ196345]
EG-48	1	Unaffliated	Clone MAT-CR-P3-G08 (83.51)[EU246116]	Hypersaline microbial mat	Uncultured candidate 06D2Y45 (84.61)[DQ330696]
EG-49	1	Betaproteobacteria	Clone ambient_uncontrolled-48 (98.79)[GU454909]	Anaerobic fermentation reactor	Ideonella sp. NS1203 (97.85)[AB636294]
EG-51	1	Alphaproteobacteria	Clone Fr1-9 (98.07)[GU208455]	Dongping Lake sediment	Nordella oligomobilis (92.81)[AF370880]
EG-52	4	Alphaproteobacteria	Clone ZWB4-16 (99.59)[FJ801213]	Zoige Alpine	Sphingomonas sp. B18

				Wetland	(97.45)[AF410927]
EG-57	1	Flavobacteriia	Clone MA-R15 (97.76)[JN038598]	Chongxi wetland soil	Flavobacterium sp. H7 (97.23)[EU109724]
EG-59	1	Cyanobacteria	Clone LI06st1 (98.62)[JF733399]	Genomic DNA	Leptolyngbya sp. FYG (92.84)[FJ933259]
EG-60	1	Flavobacteriia	Clone ASB38 (97.44)[FJ947136]	Activated sludge	Uncultured Flavobacteria CSC8 (94.36)[JN541140]
EG-61	1	Cyanobacteria	Clone EpiUMA6 (91.19)[FJ849244]	Arctic stream epilithon	Chamaesiphon subglobosus PCC 7430 (89.82)[AY170472]
EG-65	1	Alphaproteobacteria	Bacterium NTL215 (99.93)[JQ779044]	Phyllosphere	Rhizobium sp. PRNB-25 (99.93)[HQ589028]
EG-67	1	Cytophagia	Clone cuticle_2.39 (99.45)[HQ111173]	Embryonic stages	Uncultured Cytophagales 17 (99.15)[AF361198]
EG-68	1	Alphaproteobacteria	Clone 3.29h71 (95.85)[JN679095]	Membrane bioreactor	Micavibrio sp. EPC2 (88.99)[DQ186614]
EG-71	1	Actinobacteria	Clone AK4DE1_06E (93.35)[GQ397040]	Soil	Iamia sp. T2-YC6790 (92.18)[GQ369058]
EG-73	1	Sphingobacteriia	Clone 46-2_3(93.95)[FJ517715]	Epithelium	Haliscomenobacter hydrossis DSM 1100 (84.19)[NR_042316]
EG-74	2	Alphaproteobacteria	Clone DP7.3.17(87.08)[FJ612212]	Lake water	Ochrobactrum sp. TK14(86.29)[AJ550273]
EG-75	1	Gammaproteobacteria	Clone HglFeb0118m2 (88.84)[JX016688]	Marine bulk water	Saccharophagus sp. Myt-1(86.27)[AB566414]
EG-76	1	Unaffliated	Clone 654995 (88.62)[DQ404664]	Contaminated sediment	Tuber borchii symbiont b-17BO (88.45)[AF070444]
EG-77	3	Alphaproteobacteria	Clone 6-53 (99.86)[JQ923713]	Drinking water distribution system	Porphyrobacter sp. KK351(99.79)[AB033326]
EG-78	1	Betaproteobacteria	Clone 6'-72 (99.60)[JQ923814]	Drinking water distribution system	Herbaspirillum sp. CHNTR44 (95.46)[DQ337592]
EG-80	1	Gemmatimonadetes	Clone Kas174B (96.70)[EF203205]	Sediment	Gemmatimonas aurantiaca T-27 (95.45)[NR_027529]
EG-84	1	Gammaproteobacteria	Clone BJGMM-t-217 (97.39)[JQ801240]	Soil	Methylibium fulvum Gsoil 328 (96.97)[AB245357]
EG-86	8	Alphaproteobacteria	Clone Chun-s-2 (97.90)[EF632760]	Freshwater sediment	Rhodobacter sp. EMB 174 (97.91)[DQ413163]
EG-88	1	Betaproteobacteria	Clone MACA-EFT44 (97.39)[GQ500809]	Cave stream	Uncultured Methylotenera sp. sh-xj122 (95.66)[JQ327997]
EG-89	1	Alphaproteobacteria	Clone 14-95 (99.15)[JF697347]	Stream of Dianchi Lake	Rhodobacter sp. TCRI (97.06)[AB017799]
EG-90	1	Gammaproteobacteria	Clone JN11 (98.74)[JN868991]	Lake water	Dokdonella fugitiva type A3T (92.93)[AJ969432]
EG-96	1	Alphaproteobacteria	Clone SN101 (95.99)[JQ824989]	Saline-alkali soil	Sphingomonas sp. MTR-71 (95.36)[DQ898300]
EG-97	1	Alphaproteobacteria	Clone 6-45 (99.10)[JQ923705]	Drinking water distribution system	Sandarakinorhabdus limnophila so42 (99.18)[NR_043265]

EG-104	*1*	*Cytophagia*	*Uncultured Verrucomicrobia OTU-1_PBA (93.91)[JN981899]*	*Aerosols from orbal oxidation ditch*	*Uncultured Cytophaga sp.JTB251 (91.23)[AB015265]*
EG-106	*3*	*Sphingobacteriia*	*Uncultured Sphingobacteriales Holab_1.7 (97.47)[EF667893]*	*Epithelium*	*Arcicella aquatica NO-502 (97.51)[NR_029000]*
EG-110	*1*	*Deltaproteobacteria*	*Clone BN1 (88.88)[HQ190262]*	*Zhongyuan oil field*	*Uncultured Desulfocurvus sp. H-169 (85.63)[HM565023]*
EG-113	*1*	*Betaproteobacteria*	*Clone 61 (99.13)[FJ623322]*	*Aerobic sequencing batch reactor*	*Hydrogenophaga sp. AH-24 (98.79)[AB300163]*
EG-114	*1*	*Sphingobacteriia*	*Clone KWK6S.61 (95.91)[JN656817]*	*Thaw pond*	*Ferruginibacter alkalilentus HU1-GD23 (95.86)[NR_044588]*
EG-117	*1*	*Betaproteobacteria*	*Clone Q7346-HYSA (95.50)[JN391845]*	*Sludge in anoxic tank*	*Rubrivivax gelatinosus TUT3906 (95.11)[AB250625]*
EG-119	*2*	*Betaproteobacteria*	*Clone 3.29h30 (93.68)[JN679082]*	*Membrane bioreactor*	*Ralstonia solanacearum LYP (90.87)[HQ176322]*
Hyd-1	*1*	*Betaproteobacteria*	*Clone 2C229148(95.06)[EU800906]*	*Delaware Bay, NJ*	*Herbaspirillum sp. G8A1(92.75)[AJ012069]*
Hyd-4	*1*	*Gammaproteobacteria*	*Clone 6'-12 (99.80)[JQ923767]*	*Drinking water distribution system*	*Pseudomonas peli M-T-TSA_17(99.58)[JQ795777]*
Hyd-5	*1*	*Unaffliated*	*Clone ZR106(90.95)[JN713458]*	*Oral cavity subgingival plaque*	*Amphiprora paludosa C52(73.71)[FJ002240]*
Hyd-6	*1*	*Alphaproteobacteria*	*Clone GOM_WB10-31(84.86)[GQ250609]*	*Sea water column in Gulf of Mexico*	*Methylosinus sp. PW1(80.84)[AF150802]*
Hyd-8	*1*	*Verrucomicrobiae*	*Clone C2A(96.59)[DQ856525]*	*Intestinal microflora in Chinese mitten crab*	*Uncultured Verrucomicrobiaceae B07-05G(95.36)[FJ543030]*
Hyd-9	*1*	*Alphaproteobacteria*	*Sphingomonas sp. YT0136(98.76)[AB362778]*	*Genomic DNA*	*Novosphingobium sp.Van61(97.72)[HQ222277]*
Hyd-11	*4*	*Betaproteobacteria*	*Clone MSB19A(98.74)[DQ069200]*	*SA Au mine*	*Methylophilus leisingeri (98.13)[AB193725]*
Hyd-12	*1*	*Alphaproteobacteria*	*Clone MACA-OC42(91.52)[GQ500891]*	*Cave stream*	*Sphingomonas sp. MTR-71(91.41)[DQ898300]*
Hyd-14	*1*	*Flavobacteriia*	*Clone OTU-30-AW(93.42)[JQ624271]*	*Wastewater treatment plant*	*Wandonia haliotis Haldis-1(85.04)[FJ424814]*
Hyd-17	*1*	*Alphaproteobacteria*	*Clone C20(96.08)[JQ323113]*	*Cooling water system*	*Rhodobacter sp. TCRI 14(94.84)[AB017799]*
Hyd-18	*1*	*Sphingobacteriia*	*Clone SM1C08(91.67)[AF445665]*	*Genomic DNA*	*Algoriphagus sp. KJF5-15(83.59)[JQ800092]*
Hyd-20	*1*	*Alphaproteobacteria*	*Clone B1NR70D6(97.43)[AY957891]*	*Drinking water biofilm*	*Dechlorospirillum sp. DB(86.41)[AY530551]*
Hyd-22	*2*	*Alphaproteobacteria*	*Clone FGL7S_B42(97.76)[FJ437987]*	*Green Lake surface sediments*	*Rhodobacter sp. EMB 174(97.91)[DQ413163]*

Hyd-23	1	Sphingobacteriia	Clone ZBAF-R4(94.18)[HQ011389]	Biofilm in a wastewater treatment	Uncultured Bacteroidetes SW11(94.07)[AJ575719]
Hyd-30	1	Alphaproteobacteria	Clone BF2C07(97.45)[JN820204]	Ferromanganese deposit	Sphingomonas sp. MTR-71(96.25)[DQ898300]
Hyd-34	9	Alphaproteobacteria	Clone B5(99.09)[HQ697418]	Drinking water treatment	Rhodobacter sp. TUT3732(97.06)[AB251408]
Hyd-38	1	Flavobacteriia	Clone SGUS1259(97.76)[FJ202110]	Montastraea faveolata kept in aquarium	Fluviicola taffensis DSM 16823 RW262(91.88)[NR_041911]
Hyd-41	1	Armatimonadia	Clone MC20(93.20)[JN868914]	Lake water	Armatimonas rosea(92.26)[AB529679]
Hyd-42	1	Flavobacteriia	Clone ATB-LH-7198(97.22)[FJ535191]	Water of carrot washing plant	Flavobacterium aquatile DSM 1132(96.94)[NR_042495]
Hyd-43	1	Betaproteobacteria	Clone 3.29h30(98.40)[JN679082]	Membrane bioreactor	Denitratisoma oestradiolicum AcBE2-1(91.97)[NR_043249]
Hyd-45	2	Alphaproteobacteria	Clone F0813(100)[AF235997]	Genomic DNA	Sphingomonas sp. PlS3(97.25)[AJ620200]
Hyd-48	2	Betaproteobacteria	Acidovorax sp. Van23(99.26)[HQ222268]	Vanillin enrichment culture	Endosymbiont of Nilaparvata lugens M400(98.86)[JQ975881]
Hyd-49	1	Betaproteobacteria	Uncultured bacterium 0317(98.33)[AB286389]	Activated sludge	Uncultured Rhodocyclaceae Amb_16S_876(94.87)[EF018601]
Hyd-51	1	Alphaproteobacteria	Clone DUP6D09(99.64)[HQ178928]	Sediment	Sphingomonas sp. HTCC500(98.39)[AY584571]
Hyd-56	1	Betaproteobacteria	Clone JS05(98.46)[JN869110]	Lake water	Leptothrix sp. S1.1(97.19)[DQ241397]
Hyd-57	2	Gammaproteobacteria	Clone ncd1965g04c1(99.41)[JF166553]	Skin, volar forearm	Silanimonas sp. AK13(89.75)[HE573746]
Hyd-58	1	Deinococci	Clone Alchichica_AL31_2_1B_168(92.41)[JN825642]	Lake	Truepera radiovictrix TU-8(88.32)[DQ022077]
Hyd-60	1	Betaproteobacteria	Clone 1H_31(99.20)[AY546500]	Soda lake sediment	Methylophilus leisingeri(95.34)[AB193725]
Hyd-66	1	Alphaproteobacteria	Clone XZXXH70(96.28)[EU703428]	Freshwater lake	Rhodobacter sp. Cr5-50(95.18)[GU441680]
Hyd-67	1	Flavobacteriia	Bacterium SH1-3(96.17)[JQ269247]	Biofilm on antifouling paint-summer	Fluviicola taffensis DSM 16823(92.33)[NR_041911]
Hyd-69	1	Deltaproteobacteria	Clone A90(93.24)[FJ660578]	Activated sludge	Uncultured Polyangiaceae Amb_16S_1127(90.95)[EF018495]
Hyd-72	1	Betaproteobacteria	Clone glb343c(92.85)[EU978831]	Glacier ice	Uncultured Candidatus Accumulibacter sp.4.6h71(89.08)[JN679133]

Hyd-73	*1*	*Gammaproteobacteria*	*Clone B04-06F(97.56)[FJ542988]*	*High ergovaline treatment gut*	*Legionella sp. W10-070(86.21)[HE613855]*
Hyd-74	*1*	*Flavobacteriia*	*Clone 3C003394(97.63)[EU802008]*	*Chesapeake Bay, MD*	*Flavobacterium sp. MSY2(97.22)[DQ196345]*
Hyd-78	*3*	*Betaproteobacteria*	*Clone MACA-EFT44(96.67)[GQ500809]*	*Cave stream*	*Uncultured Methylotenera sp. sh-xj122(94.95)[JQ327997]*
Hyd-79	*9*	*Betaproteobacteria*	*Clone reservoir-137(99.74)[JF697518]*	*Stream of Dianchi Lake*	*Curvibacter sp. IMCC12707(98.52)[JF715440]*
Hyd-81	*1*	*Betaproteobacteria*	*Beta proteobacterium A1004(95.83)[AF236009]*	*Genomic DNA*	*Inhella inkyongensis IMCC1713(94.91)[NR_043920]*
Hyd-82	*2*	*Gammaproteobacteria*	*Cellvibrio sp. KY-YJ-1(98.66)[EF692634]*	*Genomic DNA*	*Cellvibrio fibrivorans R-4079(99.05)[NR_025420]*
Hyd-83	*1*	*Deltaproteobacteria*	*Clone FOOS7B_64(95.78)[EU431712]*	*Percolating waters*	*Bdellovibrio exovorus JSS(95.74)[EF687743]*
Hyd-87	*2*	*Alphaproteobacteria*	*Uncultured Rhizobiales bacterium Sto3-1(95.43)[AY138237]*	*human stomach biopsy*	*Prosthecomicrobium hirschii ATCC:27832(94.63)[HM037994]*
Hyd-94	*1*	*Deltaproteobacteria*	*Clone fjc-58(98.81)[JQ278802]*	*Groundwater*	*Chondromyces pediculatus Cm p51(92.64)[GU207875]*
Hyd-97	*1*	***Unaffliated***	*Clone V201-13(91.04)[HQ114028]*	*Biofilms in a full-scale vermifilter*	*Candidate division OP10(86.73)[GQ339893]*
Hyd-99	*1*	*Alphaproteobacteria*	*Clone 14-95(99.15)[JF697347]*	*Stream of Dianchi Lake*	*Rhodobacter sp. TUT3732(96.43)[AB251408]*
Hyd-109	*1*	*Alphaproteobacteria*	*Clone MN03(98.18)[JN869015]*	*Lake water*	*Rhodobacter sp. Cr5-50(95.67)[GU441680]*
Hyd-110	*1*	*Flavobacteriia*	*Clone AGS-HB2-1(95.57)[HQ403245]*	*Aerobic granular sludge*	*Wandonia haliotis Haldis-1(84.57)[FJ424814]*
Hyd-113	*1*	*Alphaproteobacteria*	*Clone J52(96.61)[HQ697493]*	*Biologically activated carbon*	*Hyphomicrobium sp. KC-IT-W2(95.57)[FJ711209]*
Hyd-118	*1*	*Alphaproteobacteria*	*Clone: UTFS-OF08-d36-35(97.51)[AB200296]*	*Activated sludge*	*Sinorhizobium meliloti GR67(86.19)[HE805562]*
Hyd-132	*1*	*Alphaproteobacteria*	*Asticcacaulis excentricus CB 48(98.24)[CP002396]*	*Pond water*	*Brevundimonas naejangsanensis HWG-A15(93.14)[JQ684238]*
Hyd-133	*1*	*Betaproteobacteria*	*Clone 8-105(94.98)[JQ923988]*	*Drinking water distribution system*	*Limnobacter thiooxidans CS-K2(94.79)[AJ289885]*
Hyd-140	*1*	*Flavobacteriia*	*Flavobacterium succinicans DSM 4001(99.25)[AM230494]*	*Genomic DNA*	*Flavobacterium sp. WB 4.3-96(97.41)[AM177633]*
Hyd-141	*1*	*Gammaproteobacteria*	*Clone FGL7S_B125(99.66)[FJ437965]*	*Green Lake surface sediments*	*Steroidobacter sp. ZUMI 37(93.93)[AB548216]*
Hyd-142	*9*	*Betaproteobacteria*	*Clone S25_1448(95.85)[EF575104]*	*Coco's Island*	*Piscinibacter aquaticus IMCC1728(95.71)[NR_043921]*
Hyd-143	*2*	*Alphaproteobacteria*	*Clone: SWB04(97.86)[AB294315]*	*Stream*	*Novosphingobium sp.*

					Van61(84.40)[HQ222277]
Hyd-145	4	Betaproteobacteria	Clone R15-71(98.07)[JF808901]	Activated sludge	Methylobacillus glycogenes DSM5685T(95.62)[FR733701]
Hyd-148	1	Cytophagia	Clone MA-R29(98.25)[JN038610]	Chongxi wetland soil	Uncultured Flexibacter sp. EfT307_D08(92.51)[GU201555]
Hyd-152	1	Gammaproteobacteria	Clone 2\SC\46(96.89)[EU340183]	Aquatic macrophytes	Dokdonella sp. TSY06(89.94)[AB663505]
wat1	1	Alphaproteobacteria	Clone sdm114(87.77)[JQ798469]	Decomposed maize straw	Rhizobium sp. PRNB-25(84.96)[HQ589028]
wat2	2	Planctomycetia	Uncultured Planctomyces sp. Joinville4(98.33)[FJ236055]	Drinking water	Planctomyces sp. Schlesner 130(87.06)[X81952]
wat5	13	Betaproteobacteria	Clone reservoir-93(99.73)[JF697474]	Stream of Dianchi Lake	Acidovorax sp. R-24667(99.60)[AM084010]
wat6	1	Actinobacteria	Clone D23(99.19)[EU234313]	Upstream of Wang Yang River	Uncultured actinobacterium STH11-15(98.85)[DQ316377]
wat7	1	Sphingobacteriia	Clone ZBAF-R4(94.25)[HQ011389]	Biofilm in a wastewater treatment	Uncultured Sphingobacteria ADK-MOe02-08(93.87)[EF520606]
wat8	1	Alphaproteobacteria	Clone FGL7S_B145(93.59)[FJ437978]	Green Lake surface sediments	Uncultured alpha proteobacterium HB125(92.32)[EF648106]
wat10	2	Deltaproteobacteria	Isolate 1112864242226(99.54)[HQ120302]	Loamy sand	Phaselicystis flava SBKo001(87.98)[NR_044523]
wat12	1	Unaffiliated	Clone S39(99.19)[JF775633]	Pond sediment	Uncultured Acidobacteria bacterium JH46(94.78)[JQ861336]
wat13	1	Sphingobacteriia	Clone sdm140(98.38)[JQ798482]	Decomposed maize straw buried	Chitinophaga ginsengisoli Gsoil 052(87.36)[NR_041375]
wat14	2	Gammaproteobacteria	Uncultured Legionella sp.2H7(97.21)[HQ003528]	Carrizo shallow lake	Legionella sp. LLAP11(96.86)[X97362]
wat18	1	Gammaproteobacteria	Clone 12TCLN056(98.86)[AB636982]	Soil	Cellvibrio gandavensis R-4069(99.19)[NR_025419]
wat19	1	Flavobacteriia	Clone SGUS1259(97.47)[FJ202110]	Montastraea faveolata	Fluviicola sp. NBRC 101268(93.52)[AB517714]
wat20	1	Alphaproteobacteria	Clone BF2C07(98.62)[JN820204]	Ferromanganese deposit	Sphingomonas sp. D31C2(95.18)[AY162145]
wat22	1	Planctomycetia	Uncultured Planctomycetales (99.17)[JN825563]	Lake	Gemmata sp. Wa1-6(99.16)[GQ889484]
wat24	1	Betaproteobacteria	Clone mv13.2(98.80)[AY424823]	Genomic DNA	Methylotenera versatilis 301(95.61)[CP002056]
wat26	3	Actinobacteria	Clone AK4DE1_06E(97.04)[GQ397040]	Soil	Iamia sp. T2-YC6790(94.56)[GQ369058]

wat27	*3*	*Alphaproteobacteria*	*Clone L65(96.88)[EU834774]*	*Lab scale EBPR-activated sludge*	*Rhodobium marinum C3(89.41)[EU445270]*
wat28	*1*	*Alphaproteobacteria*	*Clone C20(96.64)[JQ323113]*	*Cooling water system*	*Rhodobacter sp. TCRI(95.26)[AB017799]*
wat30	*1*	*Sphingobacteriia*	*Clone BF2B08(99.66)[JN820194]*	*Ferromanganese deposit*	*Haliscomenobacter hydrossis DSM 1100(98.78)[NR_042316]*
wat31	*1*	*Flavobacteriia*	*Clone SINN629(94.76)[HM128667]*	*Tanggulha Lake*	*Flavobacterium sp. 1-C-1(84.97)[GU593613]*
wat33	*1*	*Cytophagia*	*Clone Dianchi-34(98.84)[HQ324880]*	*Lake water*	*Leadbetterella byssophila DSM 17132 4M15(93.24)[NR_043233]*
wat34	*1*	*Alphaproteobacteria*	*Uncultured Novosphingobium sp.fjc-77(97.53)[JQ278820]*	*Groundwater*	*Novosphingobium naphthalenivorans TUT562(96.22)[NR_041046]*
wat36	*2*	*Flavobacteriia*	*Clone 3C003181(97.24)[EU801804]*	*Chesapeake Bay, MD*	*Uncultured Fluviicola sp. XZXXH2(96.98)[EU703456]*
wat37	*1*	*Unaffiliated*	*Clone 654995(91.06)[DQ404664]*	*Contaminated sediment*	*Tuber borchii symbiont b-17BO(90.37)[AF070444]*
wat38	*1*	*Unaffiliated*	*Uncultured candidate TM7 D116231C09(97.08)[GU180006]*	*Oil well*	*TM7 phylum sp. canine oral taxon 250 ZL096(92.81)[JN713414]*
wat43	*1*	*Alphaproteobacteria*	*Sphingopyxis sp. M2R2(99.79)[EU188914]*	*PAH polluted site*	*Sphingopyxis chilensis R9-594(99.58)[JQ660012]*
wat41	*1*	*Betaproteobacteria*	*Clone 6'-72(99.58)[JQ923814]*	*Drinking water distribution system*	*Undibacterium sp. M4-14(99.03)[HE616176]*
wat44	*1*	*Alphaproteobacteria*	*Clone ncd2177c08c1(99.16)[JF189806]*	*Skin, volar forearm*	*Devosia geojensis BD-c194(87.97)[NR_044291]*
wat45	*1*	*Alphaproteobacteria*	*Uncultured Rhodobacteraceae XZXXH70(97.13)[EU703428]*	*Freshwater lake*	*Rhodobacter sp. Cr5-50(95.81)[GU441680]*
wat46	*1*	*Alphaproteobacteria*	*Clone UTFS-OF08-d36-35(98.13)[AB200296]*	*Activated sludge*	*Micavibrio sp. EPC2(86.06)[DQ186614]*
wat48	*1*	*Unaffiliated*	*Clone Run-SP58(95.52)[AB247481]*	*Activated sludge*	*clone B1Q1E-91f-JRD28(96.38)[JF917288]*
wat50	*3*	*Flavobacteriia*	*Clone M17-10-B17(97.38)[JQ088393]*	*Crude oil reservoir*	*Flavobacteria bacterium KF030(95.98)[AB269814]*
wat52	*1*	*Flavobacteriia*	*Clone reservoir-60(98.31)[JF697441]*	*Stream of Dianchi Lake*	*Flavobacterium succinicans DSM 4002(97.01)[NR_042498]*
wat53	*8*	*Sphingobacteriia*	*Uncultured bacterium FukuS59(94.13)[AJ290042]*	*Genomic DNA*	*Uncultured Sphingobacteria ADK-MOe02-08(92.99)[EF520606]*
wat54	*1*	*Verrucomicrobiae*	*Uncultured Tolumonas sp. R40-25(99.27)[JF808981]*	*Activated sludge*	*Prosthecobacter vanneervenii 12252(96.67)[AJ966883]*
wat59	*1*	*Sphingobacteriia*	*Clone RS06101_B100(96.97)[EU101214]*	*Frasassi sulfidic cave stream biofilm*	*clone TDNP_Wbc97_206_1_92(92.75)[FJ517048]*

wat62	*1*	*Flavobacteriia*	*Clone SINN629(95.72)[HM128667]*	*Tanggulha Lake*	*Flavobacterium sp. WB 4.4-22(84.45)[AM177636]*
wat63	*1*	*Cytophagia*	*Clone reservoir-107(99.66)[JF697488]*	*Stream of Dianchi Lake*	*Uncultured Arcicella sp. XZXXH12(99.39)[EU703452]*
wat64	*3*	*Verrucomicrobiae*	*Uncultured bacterium Kas152B(98.80)[EF203197]*	*Sediment*	*Luteolibacter sp. CCTCC AB 2010415(96.67)[JN630810]*
wat65	*1*	*Planctomycetia*	*Pirellula sp. Schlesner 302(94.17)[X81942]*	*Genomic DNA*	*planctomycete str. 535(93.17)[AJ231176]*
wat67	*1*	*Flavobacteriia*	*Clone MC1_16S_47(94.44)[EU662615]*	*Floating microbial mat from sulfidic water*	*Flavobacterium hercynium WB 4.2-33(93.95)[NR_042520]*
wat70	*1*	*Betaproteobacteria*	*Uncultured bacterium FOOS7B_45(96.44)[EU431699]*	*Alcium carbonate muds*	*Herbaspirillum seropedicae Z67(96.12)[NR_029329]*
wat77	*1*	*Alphaproteobacteria*	*Clone 214(98.09)[JF828749]*	*Waste water*	*Rickettsia heilongjiangii HLJ-054(93.52)[NR_041770]*
wat78	*2*	*Alphaproteobacteria*	*Clone MP104-SW-b24(97.79)[DQ088789]*	*Crustal biotome*	*Sphingomonas sp. EZ41(96.76)[EU591707]*
wat79	*1*	*Cyanobacteria*	*Uncultured Oscillatoriales cyanobacterium LI06st1(98.48)[JF733399]*	*Genomic DNA*	*Leptolyngbya sp. FYG(92.70)[FJ933259]*
wat80	*1*	*Actinobacteria*	*Uncultured actinobacterium LT1G10(99.66)[EU117848]*	*Lake epilimnion*	*Candidatus Planktophila MWH-EgelM2-3.acI(99.66)[FJ428831]*
wat81	*1*	*Unaffiliated*	*Clone A11(88.50)[HM773452]*	*Food-wastewater treatment plant*	*Uncultured candidate division TM7 E1B-B5-114(87.31)[EF016808]*
wat83	*1*	*Gammaproteobacteria*	*Cellvibrio sp. KY-YJ-1(97.40)[EF692634]*	*Genomic DNA*	*Cellvibrio fibrivorans R-4079(97.75)[NR_025420]*
wat84	*1*	*Cyanobacteria*	*Clone EpiNCA39(97.30)[FJ849099]*	*Arctic stream epilithon*	*Oscillatoria sp. PCC 8927(90.12)[GQ351575]*
wat87	*1*	*Alphaproteobacteria*	*Clone C-78(97.62)[HQ860594]*	*Stream water*	*Rhodobacter sphaeroides 2.4.1(96.22)[NR_029215]*
wat91	*1*	*Betaproteobacteria*	*Clone 2C228284(99.07)[EU800248]*	*Delaware Bay, NJ*	*Uncultured Rhodocyclaceae Elev_16S_475(97.56)[EF019288]*
wat92	*1*	*Betaproteobacteria*	*Clone 3C003675(99.40)[EU802237]*	*Chesapeake Bay, MD*	*Uncultured Curvibacter sp. sh-xj128(99.06)[JQ327990]*
wat93	*1*	*Bacilli*	*Uncultured bacterium, SRODG012(95.33)[FM995177]*	*Genomic DNA*	*Streptococcus suis S735(79.64)[NR_036918]*
wat95	*1*	*Betaproteobacteria*	*Clone VW-86(96.58)[FM994919]*	*hot spring Mizinka*	*Curvibacter delicatus 146(96.95)[NR_028713]*
wat97	*1*	*Unaffiliated*	*Uncultured candidate Pav-SR4(98.37)[FJ482217]*	*Lake Pavin water column*	*Uncultured candidate B1Q1E-91f-EO70(98.72)[JF917287]*
wat99	*1*	*Unaffiliated*	*Uncultured Chloroflexi*	*Volcano 1, South*	*Caldilineae bacterium*

			V1B07b21(89.28)[GU369915]	Tonga Arc.	PRI 4131(80.90)[JQ733906]
wat103	2	Unaffiliated	Uncultured Chloroflexi MP-R51(87.42)[JN038723]	Chongxi wetland soil	Uncultured Chloroflexi P-R16(85.40)[JN038805]
wat104	1	Deltaproteobacteria	Clone ASB26(86.53)[FJ947130]	Activated sludge	Geobacter thiogenes K1(79.89)[NR_028775]
wat106	1	Betaproteobacteria	Clone MACA-EFT44(96.74)[GQ500809]	Bio-Sep beads in base level cave stream	Methylotenera versatilis 301(95.22)[CP002056]
wat107	1	Alphaproteobacteria	Clone TDNP_LSbc97_232_1_69(86.88)[FJ516840]	Lower sediment	Dichotomicrobium thermohalophilum DSM5002T(83.35)[FR733679]
wat108	2	Alphaproteobacteria	Alpha proteobacterium A0721(99.51)[AF235998]	Genomic DNA	Rhizobium sp. Rb122(96.82)[AB636290]
wat109	1	Actinobacteria	Clone OTU_22(93.19)[EU083500]	Hexadecane-degrading denitrifying consortium	Uncultured actinobacterium FAC31(91.75)[DQ451470]
wat111	1	Actinobacteria	Clone thermophilic_alkaline-17(98.78)[GU455257]	Anaerobic fermentation reactor	Iamia sp. T2-YC6790(93.86)[GQ369058]
wat113	1	Unaffiliated	Uncultured Chlorobi BLC04(97.34)[JN820218]	Ferromanganese deposit	Chlorobi bacterium canine oral taxon 046 1F061(81.30)[JN713214]
wat116	2	Unaffiliated	Clone p22k16ok(96.31)[FJ478864]	Undisturbed tall grass prairie	Uncultured candidate division MP-R126(95.51)[JN038791]
wat117	6	Gammaproteobacteria	Vibrio parahaemolyticus RIMD 2210633(99.67)[BA000031]	Genomic DNA	Vibrio sp. SBP(99.47)[HQ123984]
wat119	2	Unaffiliated	Clone a-86(96.45)[JX040386]	Waste water	GN02 bacterium canine oral taxon 291 ZR106(82.82)[JN713458]
wat120	2	Betaproteobacteria	Clone 6'-72(99.33)[JQ923814]	Drinking water distribution system	Herminiimonas arsenicoxydans ULPAs1(95.46)[NR_043147]
wat121	1	Unaffiliated	Uncultured candidate RB_31f(86.41)[EF123563]	Black band diseased (BBD) coral tissues	Uncultured candidate RII-TR069(83.19)[JQ580302]
wat124	1	Alphaproteobacteria	Clone PC0810_49(96.56)[JN983651]	Genomic DNA	Rhodobacter sp. CR07-62(94.96)[EU979476]
wat125	1	Alphaproteobacteria	Clone D-26(96.08)[HQ860667]	Stream water	Aquabacter spiritensis DSM9035T(89.23)[FR733686]
wat126	1	Bacilli	Clone cpw3b516(96.88)[HM921122]	Groundwater	Pirellula sp. Schlesner 302(92.25)[X81942]
wat127	1	Gammaproteobacteria	Legionella sp. W10-070(93.64)[HE613855]	Water	Legionella pneumophila Los Angelos-1(93.37)[HQ287902]
wat129	1	Betaproteobacteria	Clone DR136(96.86)[JF429180]	Primary drinking water supply	Uncultured Nitrosospira sp.

					Ba204(93.86)[JQ684443]
wat130	1	Alphaproteobacteria	Clone T1-25(98.75)[GQ487892]	Soil polluted by heavy metals	Devosia sp. R-43964(97.69)[FR691415]
wat139	1	Betaproteobacteria	Clone 3C003105(99.53)[EU801741]	Chesapeake Bay, MD	Polynucleobacter necessarius MWH-LF2-54B(99.39)[AJ964893]
wat140	2	Gammaproteobacteria	Clone KIST-JJY048(91.15)[EF659421]	Suspended sludge from anammox reactor	Legionella sp. OUB41(89.83)[AB058918]
wat141	1	Sphingobacteriia	Flexibacter sancti IFO 15057(98.71)[AB078066]	Genomic DNA	Chitinophaga sancti IFO 15057(98.71)[NR_040917]
wat143	2	Alphaproteobacteria	Clone: SWB04(98.50)[AB294315]	Stream	Novosphingobium hassiacum W-51(84.52)[NR_028962]
wat145	2	Gammaproteobacteria	Clone AW67(99.39)[JN868837]	Lake water	Pseudomonas sp. N1(98.99)[EU275166]
wat147	1	Betaproteobacteria	Clone 3C003190(99.93)[EU801813]	Chesapeake Bay, MD	Methylotenera versatilis 301(97.93)[CP002056]
wat148	1	Gammaproteobacteria	Clone AW55(98.87)[JN868834]	Lake water	Silanimonas sp. AK13(95.17)[HE573746]
wat149	1	Sphingobacteriia	Chitinophaga sp. SaZS3(99.73)[JQ806450]	Genomic DNA	Flexibacter sancti:IFO 16034(98.57)[AB078068]
